# Social value of pathology: adapting primary health care to reduce stress and social anxiety in college students exposed to social distancing

**DOI:** 10.3389/fpsyg.2023.1143221

**Published:** 2023-06-13

**Authors:** Ionel N. Sava

**Affiliations:** Department of Sociology, University of Bucharest, Bucharest, Romania

**Keywords:** healthcare, college students, online education, stress and anxiety, pathology introduction

## Abstract

This article examined the impact of online education on the wellbeing and emotional health of college students. It considered the social value of stress and anxiety pathology as “normal” side effects throughout the COVID-19 lockdown. Factors appropriate for educational technology were selected and submitted for evaluation to a sample of 114 college students in a semi-structured questionnaire. This research found that educational content and delivery methods, as well as increased homework and time spent online, have potentially contributed to heightened levels of stress, depression, and social anxiety disorder among approximately one-third of students who have engaged in digital learning. The results also prove that young people were particularly susceptible to stress and social anxiety disorders during the lockdown, making them one of the most vulnerable social groups. To enhance the educational experience, several suggestions have been proposed, including adapting educational content, expanding Internet accessibility, providing appropriate homework, and adjusting schedules to accommodate students' educational capabilities. Voluntary routine mental health assessments of students, teachers, and staff and customized online counseling for vulnerable subjects are recommended as primary health care measures during online education.

## 1. Introduction

Mental illnesses were responsible for causing up to a 27% increase in the prevalence of anxiety and depression worldwide during the COVID-19 pandemic. The WHO estimated that successive lockdowns led to a 27.6% increase in cases of major depressive disorder (MDD) and a 25.6% increase in cases of anxiety disorders (AD) (WHO, 2022).

Of the many types of mental disorders, depression and anxiety were prevalent disorders among college students exposed to social distancing during the COVID-19 pandemic.

As such, anxiety and depression were declared to be prevalent by more than half of the participants of a sample of 1,173 students from a university in the North of England with PHQ-9 levels above the clinical cutoffs (Chen and Lucock, 2022). According to the data collected from a study at Texas A&M University using the Patient Health Questionnaire-9 and the General Anxiety Disorder-7 (GAD-7), of the 2,031 participants, 48.14% showed a moderate-to-severe level of depression, while 38.48% showed a moderate-to-severe level of anxiety, and 18.04% reported experiencing suicidal thoughts (Wang et al., 2020). A nationwide cross-sectional survey study involving 821,218 college students in China found mental health problems among 45% of participants (Ma et al., 2020). Similarly, the GAD-7 scale measured a mental health risk value of 38.4% in a sample of 1,961 university students in Poland (Rogowska et al., 2021).

Similar results were reported for smaller samples. In Australia, a stress and anxiety study in a sample involving 109 college students showed that, if weighed against anxiety (GAD-7) and depression (PHQ-9), social anxiety presented a tougher correlation with a predilection for online social interaction. However, depression and anxiety had lower values if daily Internet use did not exceed 4 h (Hutchins et al., 2021). In Romania, social distancing appeared to cause stress and anxiety in up to 48% of a sample of 100 students that were surveyed the first week after returning to in-person education (Sava, 2022).

However, for face-to-face social network interaction, research revealed that up to 15% of university students showed clinically relevant levels of depressive symptoms, while 29% of them showed symptoms of social anxiety, as they usually avoided in-person relationships (Elmer and Stadtfeld, 2020). Clinical studies have also found that depression and anxiety diminished in subjects with greater online communication (Stuart, 2021). On a larger scale, it confirmed previous research that pointed out that cultural and social factors normalize individual anxiety within the youth population (Mikolajczyk, 2008).

Up to one-third of young people aged 15 to 29 exhibit social anxiety symptoms and prefer Internet use as a social interaction avoidance strategy. This behavior has become a “normalized” aspect of social life. It indicates that a similar percentage of college students surveyed in various social and cultural contexts, who reported experiencing stress and anxiety during the COVID-19 lockdown, would perceive such feelings as “normal” or anticipated pathology. However, it is important to note that this research does not provide concrete evidence for the decrease in sharing tendencies among previously non-anxious individuals during online education.

The evidence from this research suggests that Internet use is not clearly correlated with increased social anxiety disorder despite the fact that individuals with social anxiety symptoms prefer online interactions. In such cases, the Internet is viewed as a coping mechanism rather than a cause of anxiety. It is possible that people with introverted orientation may experience some exacerbation of anxiety, but the Internet paradox does not usually lead to a number of individuals with anxiety disorder-related symptoms (Kraut et al., 1998).

Therefore, negative cognitive beliefs predisposing anxious people to avoid unpleasant face-to-face social encounters (Clark and Wells, 1995) have to be reconciled with the positive results of online social interaction (Kraut et al., 2002; Hutchins et al., 2021). Using this perspective, it should eventually be explained to what degree social distancing as a public health measure and/or communication technology use as a substitute for in-person education are still responsible for up to 15% of students' stress and social anxiety out of the 45% overall value reported during the pandemic. Understanding this relationship can provide valuable insights to healthcare providers regarding the specific counseling needs of students, teachers, and staff.

## 2. Materials and methods

Sickness is intrinsically linked to the deterioration of social conditions. It represents a deviation from what institutionalized human response claims to be “normal.” In other words, “some sort of pathology exists … whenever deviant behavior appears,” for which social confinement is necessary.

For this reason, “the critical variable in the study of [health] deviance is the social *audience* rather than the individual *person*, since it is the audience which eventually decides whether or not any given action or actions will become a visible case of deviation” [Erikson, 2013 (1962)]. Illness is a deviation similar to crime, for which, as Durkheim stated, collective action to treat it is useful to the society as “a factor of public health, an integrative element in any health society” (Jones, 1986). The pandemic is cast as a sort of social anomie, a collective ill-health that needs public intervention.

As such, pathology has social value as it predicts the risk of illness, pinpoints vulnerable individuals, and informs public measures that need to be considered. It also implies that pathology is a “normal” occurrence during times of public sickness and that customized healthcare policies are needed.

Public health policies implemented to address the COVID-19 pandemic (as decided by audiences) varied from zero infection acceptance to social distancing and lockdowns, hospital treatment, mass vaccination, or a combination of these strategies. Zero public action was exceptional or localized. Therefore, social distancing, vaccination, medical treatment, and lockdowns became “normal” procedures intended to protect people from both physical and psychological health deviations. The prevailing social logic of illness is to limit its collective consequences as much as possible. Romania engaged in active health measures during the COVID-19 pandemic, including lockdown, vaccination, and hospital treatment for those in need.

Nevertheless, such measures are expected to mitigate the impact of illness and reduce individual stress and anxiety. As such, pathology related to depression, social anxiety disorder, and suicide may still emerge as residual outcomes. The WHO reported a 26% increase in mental disorders during the pandemic. College students were among the most exposed groups to the COVID-19 pandemic's side effects. One should notice that online counseling was occasionally used as a normal/necessary healthcare procedure during the pandemic. The consequences are reflected in the stress and social anxiety that surged during the lockdowns.

Therefore, this research highlights the pathology's social value in increasing public awareness of the vulnerabilities faced by young people during pandemics. It underscores the importance of implementing health measures that address youth vulnerabilities in the medium and long run.

To measure the impact of public policy on the wellbeing and mental health of college students during the lockdown and digital learning, educational technology factors were selected and submitted to Romanian college students' online evaluation in a semi-structured questionnaire. Alongside educational factors, a number of questions checked for situational (facilitating conditions) and interactional factors such as perceived abilities to accomplish educational tasks under stress as well as worried about pandemics, individual anxiety, supported by family, missing friends and colleagues, and intent to leave university. Considering the public polarization that occurred during the pandemic, it is important to assess the pros and cons of public measures, including those implemented in Romania.

The measurement methodology mainly used three-point Likert scales, which improved polar points such enabling respondents to express their agreement and disagreement, as well as their likes and dislikes. The methodology also reinforced the inclusion of a neutral position. To ensure the reliability of the results, the Pearson Chi-square test of independence was conducted to verify mutual exclusiveness among the responses. This statistical test helped validate the relationship between variables and determine if there were any significant associations or dependencies among them.

## 3. Selecting measurement factors and subjects

During successive COVID-19 lockdowns in Romania, parents and pupils were confined at home for as long as 2 years. Physical and emotional circumstances such as worried about pandemics, sharing a room and computer with siblings, and too much time online are factors that affect online education. Trust in one's own abilities to perform distance education (self-efficacy), perceived relevance (motivation), and satisfaction with content (affect) are considered situational factors (Kemp et al., 2019). Social interactivity factors refer to student-teacher interactions, relationships with peers, missing colleagues, good feedback from professors and missing outdoor activities. Finally, facilitating, situational, and social interactivity factors are, in various degrees, related to the stress and social anxiety that individuals encounter during online sessions.

For this report, I selected the following factors: (a) comfortable and safe at home, (b) worried about pandemics, (c) perceived stress and anxiety, (d) lack of human (face-to-face) interactions, (e) missing colleagues, (f) time pressure, (g) good feedback from professors, and (h) abandon studies.

The survey measured students' individual experiences. A number of open-ended questions were also submitted for their consideration. The received answers were coded in fields according to the items above. Table 1 displays quantitative measurements. Table 2 presents qualitative data.

**Table 1:** Distribution of perceived COVID-19 disruption^*^.

**Frequency by sample item**	**More/same as before/less**				**n**
	**UNIBUC**	**UTV**	**Aggregate**	χ2^**^	
Worried about pandemics	38/1/10	32/0/12	70/1/22	22.64	93
Stress	25/4/20	24/5/21	49/9/41	27.15	99
Missing most (colleagues)	34/11/5	30/1/19	64/12/24	44.48	100
Feedback from professors	15/25/10	5/41/1	20/66/11	53.84	97
Affect (satisfied with content)	3/31/15	2/24/24	5/55/38	39.58	98
Time pressure	42/2/1	46/1/0	88/3/1	160,88	92
Abandon studies	5/25/20	12/4/31	17/29/51	18.39	97

^*^Data collected in March 2022 at the end of the COVID-19 lockdown.

^**^The Chi-square test significance level is α = 0.05, and the critical value is χ^2^= 5.99.

**Table 2:** Qualitative emotional data display.

**Field**	**Category**	**Students (** ***n*** **)**		
		**UNIBUC**	**UVT**	**Total**
Safety and family	Comfortable and safer at home	28	32	60
	Supported by family	40	42	82
	Protected against Covid-19	21	18	39
Circumstantial	Worried about pandemics	37	32	69
	Too much time online	22	16	38
	Missing open air activities	11	20	31
Educational	Impersonal teaching	6	9	11
	Too busy schedule	23	28	51
	Missing study trips/internships	8	16	24
	Good feedback from professors	9	6	15
Emotional	Missing friends and colleagues	24	30	54
	Stress/difficulties to focus	14	18	32
	Lack of human interaction	23	27	50
	Increased assignments	18	24	42

^*^Data collected in March 2022 at the end of the COVID-19 lockdown.

A total of 114 participants were asked to provide consent and fill out the questionnaire. The selected 100 students were extracted from the University of Bucharest and the Western University of Timisoara. Demographic data reflect main population characteristics regarding gender distribution (65 women and 35 men) and age ranking (from 19 to 26 years old with a mean of 22.6 for the selected sample of *n* = 100). Female ascendancy is specific to the social and humanistic studies of the selected universities. Respondents were not asked about their racial or ethnic identities, and they received no financial incentive to participate. They provided answers under conditions of anonymity, and no apparent bias was introduced. All participants attended at least two semesters of online education (one academic year).

## 4. Results and observations

As reported by the participants, with the COVID-19 lockdown and the switch to online education, a number of circumstantial, educational, and emotional-specific outcomes occurred. Worried about pandemics ranked first (69% value), followed by social anxiety (missing colleagues by 64%), stress (49%), time pressure due to online activities (almost 90% of students complained they spent too much time in front of the computer), and, finally, intentions to abandon studies (18%, see Table 1).

However, the same category factors returned certain positive feedback for supported by family (82%), comfortable and safer at home (60%), and protected against COVID-19 (39%, see Table 2). These factors also measured wellbeing during the pandemic.

Educational factors measurement returned several emotional health challenges, such as too much time spent on online schooling (38%), difficulties associated with focusing (32%), impersonal teaching (11%), and losing feedback from instructors (15%). Emotional challenges multiplied regarding social anxiety (64% of subjects reported missing friends and colleagues) and a lack of human interaction (50%). Female students reported a certain emotional overload. Nevertheless, to mitigate the negative impact of social distancing, online education increased the homework load, as students mentioned in both closed- (48%) and open-ended questions (42%). The results were contrary to expectations. Almost half of the students complained about increased assignments online, and it seems to be one of the main sources of social anxiety. The more time subjects spend online, the less capacity they have to focus, ultimately hindering their effectiveness in achieving educational goals.

## 5. Discussion

Soon after the March 2020 lockdown, with the online education switch, one research article pointed out that “students reported stress, anxiety, being worried about getting sick (COVID-19), and changes in their mental health” (Aguilera-Hermida, 2020). Since Internet use has not been proven to be directly responsible for the rise in social anxiety (Kraut et al., 2002; Hutchins et al., 2021), it is necessary to explore other emotional challenges that could potentially be accountable (Elmer and Stadtfeld, 2020).

During successive lockdowns, universities created ad-hoc educational fields (digital social arenas) using computer communication technologies. The improvised solutions serve as contingency substitutes for in-person education, offering a temporary alternative. Digital educational platforms play a role in facilitating social interaction (facilitation), which is similar to the support provided by modern medical advocacy (Smith and Stewart, 2017). “Patients” felt safe at home (60%) and received family support (82%) while still taking part in their social and educational networks' activities.

However, in a variety of social and cultural contexts, almost half of the college students reported experiencing increased levels of stress and social anxiety during the online education period (Wang et al., 2020). Unfortunately, there was a lack of customized healthcare policies specifically aimed at addressing the surge in stress and social anxiety among college students. Consequently, irregular outcomes were eventually reported.

On the one hand, as Yen et al. (2012) also demonstrated well before the pandemic that “social anxiety is lower during online interaction than during face-to-face interaction, especially in subjects with high social anxiety [and] depression.” Qualitative statements confirmed better social interaction online, as one female student stated, “I did not attend classes before, as I was anxious and shy, so online was better, and my relationship with professors had improved.” One of her colleagues also stated that “At the beginning, I felt as in a permanent vacation, being able to stay all day with my family, and I felt safe from the virus.”

On the other hand, even though students were at home, they often found themselves becoming inattentive as they spent more time on their regular educational tasks compared to traditional in-person education. One student at the University of Bucharest mentioned that the “Pandemic stole 2 years of my life” because online interaction consumed most of his/her time. A similar answer was mentioned by another student: “It was impersonal, and I was away from colleagues and professors.” Such idiosyncrasies offer genuine symptoms of stress and anxiety that are associated with online social interaction indeed.

Subjects who constantly worried about pandemics (70%), who were missing colleagues (64%), and who experienced a lack of human interactions (50%) during online sessions were the first to report stress, resulting in an increase in social anxiety. There is no research to replicate similar social distancing conditions, but one could estimate that stress and anxiety can reach a mass scale in the absence of Internet service for as long as 2 years.

On the other hand, reasons for subjects reporting time pressure (80%), dissatisfaction with content (30%), and difficulties focusing (32%) were eventually related to the use of communication technologies. Nevertheless, they showed a positive attitude toward technology use (60%) and good motivation (40%) during online sessions (Sava, 2022).

This report revealed that content received, delivery methods, class assignments, and time spent online are responsible for a significant portion of the increased stress and anxiety experienced by college students during the COVID-19 lockdown. The majority of subjects in this research complained about spending too much time online. Moreover, 37% of students mentioned experiencing less satisfaction with content, while 42% perceived that increased homework was not justified. A number of subjects (18%) considered abandoning university. All of these results were recorded against 48% technology acceptance and 60% favorable attitudes toward Internet technology use. The paradox is that online education has side effects that are not due to technology use.

(Kraut et al., 1998) called it the “Internet paradox”, i.e., a decline in the size of social circles and an increase in depression and loneliness among individuals who spend time online. Hutchins et al. (2021) proved that depression and anxiety had lower values if daily Internet use did not exceed a certain amount of time. There is no paradox if the time frame and content delivered are in the right range.

This research discovered that up to one-third of people aged 15 to 29 are susceptible to social anxiety symptoms. Online education is more of a coping mechanism for this group. Nevertheless, there is a surge of 15% in stress and social anxiety, which is associated with the COVID-19 pandemic. This study fills the research gap by showing that mental disorders increase as a side effect of online time and the delivery methods used during online education.

Digital learning served as a substitute for in-person education, yet online delivery only partially fulfilled students' expectations. The implications of this study are twofold: first, online education proved to be beneficial for young subjects with a predisposition for social anxiety and, second, it served as a compensatory measure for managing stress and anxiety in subjects exposed to successive lockdowns and public health risks. Further research is needed to strengthen and consolidate this finding within the negative cognitive theory debate. It appears reasonable to advocate for the inclusion of healthcare policies that address mental disorders associated with digital learning. For a certain number of students, online counseling seems necessary, just as online learning is for others.

## 6. Conclusions

The interruption of in-person education confirmed communication technology's important complementary role as a digital substitute for human interaction. Regarding the stress and social anxiety that presumably escalated during COVID-19, this report found no explicit evidence that communication technology was responsible. Other things being equal, up to one-third of young people aged 15 to 29 prefer to use Internet communication technologies to avoid face-to-face interaction. The remaining part reported that stress and social anxiety disorders were considered secondary effects of healthcare measures to confine the pandemic and improper technology use. This research found that content and delivery methods, along with increased homework and time spent online, can potentially rise individual pathologies of stress, depression, and social anxiety disorder for up to one-third of students exposed to digital learning.

It is up to various cultural and social contexts to diminish this subsidiarity. Healthcare policies should be developed alongside new educational apps and policies. We propose designing applications to enhance online education, making delivery routines more tailored to students' needs, adapting educational content for online and smartphone use, increasing Internet accessibility, ensuring appropriate homework, and adjusting schedules to accommodate students' educational capabilities. Online programs aimed at reducing stress and social anxiety are necessary educational programs. Finally, this research recommends using online education as a complement to in-person education, with the latter remaining the core of higher education.

## Data availability statement

The original contributions presented in the study are included in the article/supplementary material, further inquiries can be directed to the corresponding author.

## Ethics statement

The studies involving human participants were reviewed and approved by Institutional Review Board Statement: University of Bucharest # SAS 562-2022. The patients/participants provided their written informed consent to participate in this study.

## Author contributions

The author confirms being the sole contributor of this work and has approved it for publication.
